# Modelling the risk of transfusion transmission from travelling donors

**DOI:** 10.1186/s12879-016-1452-z

**Published:** 2016-04-01

**Authors:** Tonderai Mapako, Welling Oei, Marinus van Hulst, Mirjam E. Kretzschmar, Mart P. Janssen

**Affiliations:** 1 Unit of PharmacoEpidemiology & PharmacoEconomics (PE2), Department of Pharmacy, University of Groningen, Groningen, The Netherlands; 2 National Blood Service Zimbabwe, Harare, Zimbabwe; 3 Transfusion Technology Assessment Unit, Sanquin Research, Amsterdam, The Netherlands; 4 Julius Center for Health Sciences and Primary Care, University Medical Centre Utrecht, Utrecht, The Netherlands; 5 Department of Clinical Pharmacy and Toxicology, Martini Hospital, Groningen, The Netherlands; 6 National Institute for Public Health and the Environment, Bilthoven, The Netherlands; 7 Center for Infectious Disease Control, National Institute for Public Health and the Environment, Bilthoven, The Netherlands

**Keywords:** Travellers’ risk, Transmission risk, Emerging infectious diseases, Blood transfusion

## Abstract

**Background:**

The EUFRAT (European Up-Front Risk Assessment Tool) was developed as an online risk assessment tool (http://eufrattool.ecdc.europa.eu) to help decision-makers assess the transmission risk of emerging infectious diseases (EID) through blood transfusion. The aim of this study is to extend the methodology developed in the EUFRAT project to quantify the transfusion transmission (TT) risk from travelling donors.

**Methods:**

A generic model for estimating the TT risk from a group of travelling donors that visited an EID risk area was developed. In addition, the new model distinguishes projected future transmissions from those that have already occurred. As an illustration the model was applied to the outbreaks of chikungunya in Italy in 2007 and Q fever in the Netherlands in 2007–2009.

**Results:**

Formulas for calculating the travelling donors’ TT risk were derived. For the chikungunya outbreak in Italy an early intervention (at the end of week 7 after the start of the outbreak, so after only 19 % of all cases) would have been required to prevent only 41 % of all expected transmissions at that time. For Q fever, in which the transmission of chronic Q fever is considered, even at the end of the third annual outbreak’s peak 47 % of all (chronic) Q fever transmissions could still be prevented.

**Conclusions:**

The updated model allows estimation of the infection transmission risk from travelling donors. In combination with the distinction between past and future transmissions, these estimates provide valuable information to support decisions concerning communication with the public and/or the implementation of safety interventions.

**Electronic supplementary material:**

The online version of this article (doi:10.1186/s12879-016-1452-z) contains supplementary material, which is available to authorized users.

## Background

Trends in globalisation have brought increased blood safety concerns as population members (and therefore also blood donors) have become highly mobile. This presents challenges for blood safety when travellers go to areas in which there is ongoing transmission of infectious diseases (either endemic or epidemic) and import infections to their home countries. Blood safety concerns for emerging infectious diseases (EID) are identified and documented extensively in the literature [1–4]. Currently available travellers’ risk models focus mainly on the probability of travellers acquiring an infection when visiting high-risk areas [5–7], but do not link this information directly to its implications for the risk of transmission by (blood) donors.

Several blood establishments have travel risk policies for donors who report having visited EID risk areas [2, 4, 6, 8–10]. Variation in existing policies may be an indication of the absence of a common understanding and perception of the blood safety risk posed by travellers in their respective home countries. The EUFRAT (European Up-Front Risk Assessment Tool) project [2] developed and launched an online risk assessment tool [11] to help decision-makers assess the EID risk in order to guide informed decisions concerning an appropriate course of action. The current tool, however, offers only limited opportunities for extensively analysing the transfusion transmission (TT) risk from travelling donors.

The aim of this study was to extend EUFRAT to characterise and model the risk of disease transmission from travelling donors to blood safety. We propose generic formulas for calculating the risk of transmission taking into account transfusions that have already occurred by the time of observation (past transmissions), and the projected (future) transmissions from travelling donors. The method was applied to the chikungunya outbreak in Italy [2, 10] and the Q fever outbreak in the Netherlands [12, 13] that have previously been analysed using EUFRAT for the TT risk among the local residents. We chose the chikungunya and Q fever examples to illustrate risk estimates for a disease with a short and a long infectious period.

## Methods

### Deriving the travellers risk model

For the quantification of the risk of infected donations from travellers who visited an EID risk area, a number of definitions are required. These relate to (1) the transmission in the EID risk area (number of infections notified, duration of observation, population size), (2) the infection (duration of the infectious period, proportion of symptomatic and chronic infections), and (3) the travelling donor population (number of travellers, duration of visits, donation frequency). Next, based on these definitions, formulas for the expected number of transmissions by blood transfusion from travellers were derived. A distinction is made between transmissions that have already occurred by the end of the observed outbreak (past transmissions) and for transmissions yet to occur (future transmissions).

**Fig. 1:**
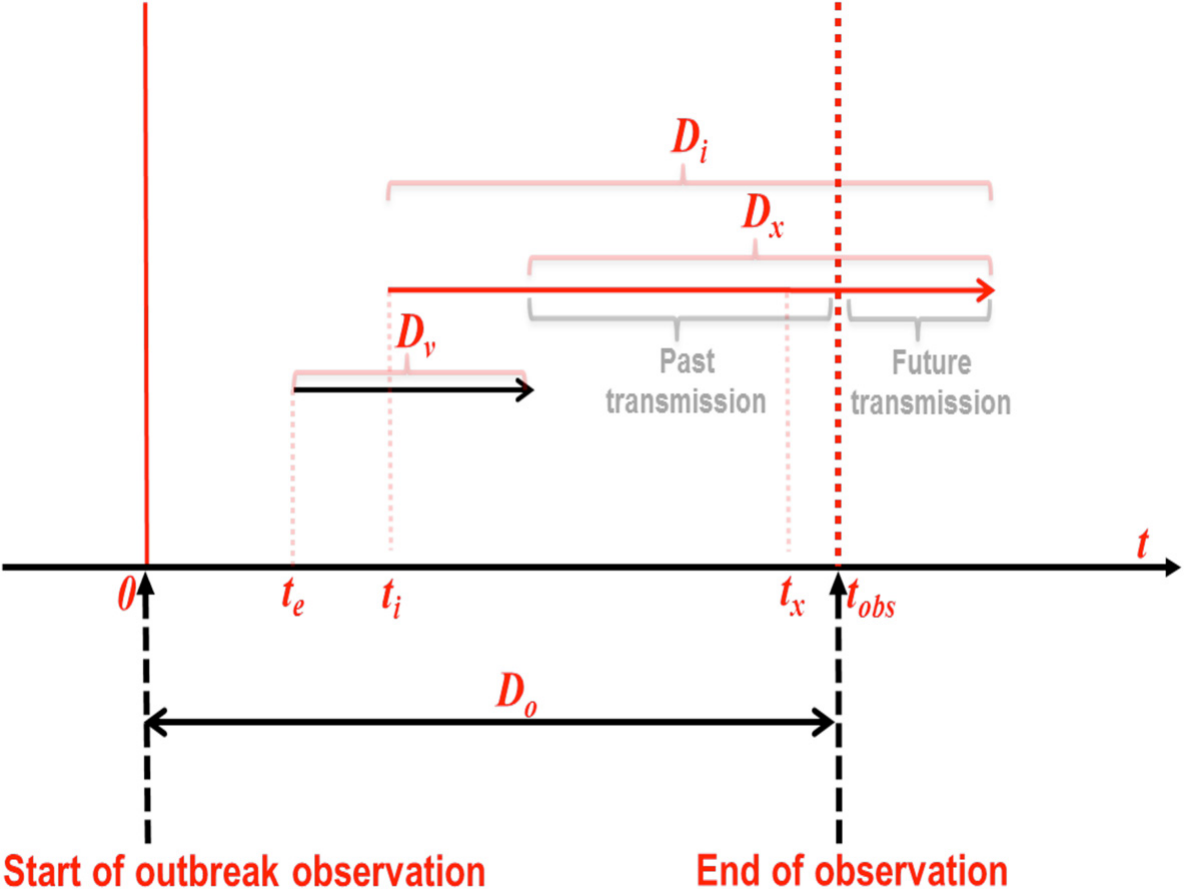
Modelling travellers’ risk when visiting a risk area. The key features to note are that travellers’ exposure varies depending on the time of entry (***t***_***e***_) in relation to the start and end of the observation (***t*** **= 0,** ***t*** **=** ***t***_***obs***_ **=** ***D***_***o***_). Transmission risk is further affected by the time of getting infected (***t***_***i***_), and the time of donating (***t***_***x***_) after the travellers’ return to their home country. Transmission will only occur if donation takes place within the remaining infectious period (***D***_***x***_). Other factors considered are the duration of the visit ***D***_***v***_, the duration of infectivity ***D***_***i***_, and the duration of the observation ***D***_***o***_. Returning donors who have already donated at the end of observation ***t***_***obs***_ (past transmissions) can obviously not be prevented. Transmissions that are yet to occur (future transmissions) can be prevented by implementing additional safety interventions

The model of a travelling donor entering a risk area, his exposure, infection and his infectious period is illustrated in Fig. 1. A summary of parameters used in the model is given in Table 1. Details on the derivation of the formulas can be found in the Additional file 1.

**Table 1:** Description of model parameters and their values used in estimating travellers transmission risks for the respective chikungunya [2, 10] and Q fever [1, 3] outbreaks

Symbol	Dimension	Description	Chikungunya	Q fever
*I*	-	Number of infections: number of infections in the risk area.	247	837
*N*	-	Population size: the size of the local population in the risk area.	3,977,508	55,725
*D*_o_ = *t*_*obs*_	Time	Duration of the observation: the time from the 1^st^ day of reported cases until the day of the last reported case; or for a series of observations: the time from the start until the end of each observation period.	105 days (15 weeks)	1050 days (35 months)
$\lambda =\frac{I}{N{D}_0}$	1/time	Incidence rate: the rate of infection accrual in the risk area.	5.9 × 10^−7^ per day	1.4 × 10^−5^ per day
*D*_*i*_	Time	Duration of infectious period: the time in which a traveller is infectious.	8 (1–12) days	Acute: 14 (10–17) days Chronic: 12 (3–21) months
*D*_*x*_	Time	Duration of infectious donation period: the time in which a traveller might give blood transfusion during his infectious period after returning home.		
*p*_*d*_	-	Proportion of donors: the proportion of donors among the general population.	3.53%^a^	2.37%^a^
*τ* = *p*_*d*_*f*_*v*_	1/time	Rate of donors visiting the risk area: this is calculated from a proportion of donors (*p*_*d*_) among the number of visitors to the risk area per unit time (*f*_*v*_).	0.35 donors/day^a^	0.24 donors/day^a^
$\varphi =\frac{1}{D_d}$	1/time	Donation rate: number of donations per unit time, this depends on the inter-donation interval (*D*_*d*_), i.e. time in between subsequent donations by donors.	0.005 per day^a^ (*D*_*d*_ = 215)	0.005 per day^a^ (*D*_*d*_ = 215)
*D*_*v*_	Time	Duration of visit: length of stay of visitors in the risk area.	7 days^a^	14 days^a^
*t*	Time	Time: the time since the start of the observation.		
*t*_*e*_	Time	Time of donor entry: the time at which a travelling donor arrives in the risk area within the observation period.		
*t*_*i*_	Time	Time of infectivity: the time at which the travelling donor is presumed to obtain an infection.		
*t*_*x*_	Time	Time of donation: time at which an individual donor is assumed to deliver an infected donation.		
*N*_*v*_	-	Number of transmissions - from travelling donors after returning to their home country.		
*N*_*vf*_	-	Number of future transmissions – from travelling donors after the end of the so far observed transmission, so after time point *t*_*obs*_.		

^a^Fictive parameter values or assumptions

We consider the situation in which a traveller enters an area with ongoing transmission of an infectious disease (which we refer to as “risk area”) at time *t*_*e*_, where he remains for a time period *D*_*v*_ after which he returns to his home country. If he becomes infected he will remain infectious for a period *D*_*i*_, and in case he donates within this time period upon returning home, he is expected to transmit the infection acquired. Thus, the exposure of the traveller begins upon entering the risk area at *t*_*e*_, and lasts for a period *D*_*v*_. Because we are interested in the donations made by the traveller after the end of his visit *D*_*v*_, the time being infectious while able to donate is defined as the time interval from *t*_*e*_ + *D*_*v*_ to *t*_*i*_ + *D*_*i*_. Let us call this interval *D*_*x*_(=*t*_*i*_ + *D*_*i*_ − *t*_*e*_ − *D*_*v*_), which is relevant for transfusion transmission. All time intervals are presumed to be non-negative.

We assume that transmission in the area during the risk assessment can be characterised by a constant incidence rate $\lambda =\frac{I}{ND_o}$, where *I* is the number of infections notified in the time interval *D*_*o*_ corrected for underreporting, *N* is the size of the population at risk, and *D*_*o*_ is the period of transmissions observed. For a transmission where the incidence rate varies over time, the risk can be estimated for smaller *D*_*o*_ intervals with varying incidence rates. These varying incidence rates can be modelled as a series of individual small outbreaks of which the results can be subsequently aggregated. We next assume that the incidence rate can be interpreted as a proxy for the force of infection to which a susceptible traveller is exposed, that a donor will be infected only once during his visit, that the infection risk is proportional to the duration of stay in the risk area, that *τ* is the number of donors per unit time travelling to the risk area, that *φ* is the donation rate (which is one divided by the length of the inter-donation interval *D*_*d*_), and that each donation made by each infected donor upon returning home results in one infected recipient. The total number of transmissions from travelling donors (*N*_*V*_) is (in case *D*_*i*_ *≥* *D*_*v*_) equal to:

1 $$ \begin{array}{ll}{N}_v\hfill & ={\displaystyle {\int}_{t_e=-{D}_v}^{D_o}}{\displaystyle {\int}_{t_i= max\left({t}_e,0\right)}^{min\left({\mathrm{D}}_{\mathrm{o}}, \kern0.5em {\mathrm{t}}_e+{D}_v\right)}}{\displaystyle {\int}_{t_x={t}_e+{D}_v}^{t_i+{D}_i}}\tau\;\lambda\;\varphi\;{dt}_x\;{dt}_i{dt}_e\hfill \\ {}\hfill & =\frac{1}{2}{D}_o\tau \lambda \varphi \left(2{D}_i-{D}_v\right){D}_v=\frac{D_v\tau }{N}I\varphi \left({D}_i-\frac{1}{2}{D}_v\right)\hfill \end{array} $$

The number of transmissions per infected donor would generally be proportional to the donation rate (*φ*) and the duration of the infectious period (*D*_*i*_). For travelling donors however, half the duration of the visiting time is subtracted from the infectious period. This makes sense as donors will not be able to donate during their visit to the risk area and therefore the infectious period of a visiting donor will on average be reduced by half the visiting time.

For those cases where *t*_*e*_ + *D*_*v*_ < *t*_*obs*_, a fraction of the total number of infected donations are expected to have already been made at the time of observation *t*_*obs*_. Such transmissions cannot be prevented any more. The remaining fraction, however, can potentially be prevented by an intervention in the blood supply. This remaining fraction can be determined by splitting the formula into transmissions occurring before *t*_*obs*_ (past), and transmissions after *t*_*obs*_ (=D_o_) (future):

$$ {N}_v={\displaystyle {\int}_{t_e=-{D}_v}^{D_o}}{\displaystyle {\int}_{t_i= max\left({t}_e,0\right)}^{m in\left({D}_o, \kern0.5em {t}_e+{D}_v\right)}}\tau \lambda \varphi \left({D}_o-{t}_e-{D}_v\right){dt}_i{dt}_e+{\displaystyle {\int}_{t_e=-{D}_v}^{D_o}}{\displaystyle {\int}_{t_i= max\left({t}_e,0\right)}^{min\left({D}_o, \kern0.5em {t}_e+{D}_v\right)}}\tau \lambda \varphi \left({t}_i+{D}_i-{D}_o\right){dt}_i{dt}_e $$

Thus, the number of future transmissions from travelling donors (*N*_*vf*_) expected at *t*_*obs*_ for *t*_*e*_ + *D*_*v*_ < *t*_*obs*_ can be calculated as:

2 $$ {N}_{vf}={\displaystyle {\int}_{t_e=-{D}_v}^{D_o}}{\displaystyle {\int}_{t_i= max\left({t}_e,\ 0\right)}^{min\left({D}_o, \kern0.5em {t}_e+{D}_v\right)}}\tau \lambda \varphi \left({t}_i+{D}_i-{D}_o\right){dt}_i{dt}_e $$

For situations in which the infectious period is longer than the duration of stay (*D*_*i*_ ≥ *D*_*v*_), the number of future transmissions (so transmissions that will occur after *t*_*obs*_) is equal to:

3 $$ {N}_{vf}=\left\{\begin{array}{cc}\hfill {D}_0\ge {D}_i\ge {D}_v:\hfill & \hfill \frac{\tau I\varphi \left(3{D}_i^2-{D_v}^2\right){D}_v}{6{ND}_0}\hfill \\ {}\hfill {D}_i\ge {D}_0\ge {D}_v:\hfill & \hfill \frac{\tau I\varphi \left(6{D}_0{D}_i-3{D_0}^2-{D_v}^2\right){D}_v}{6{ND}_0}\hfill \\ {}\hfill\ {D}_i\ge {D}_v\ge {D}_0:\hfill & \hfill \frac{\tau I\varphi \left(6{D}_v{D}_i-3{D_v}^2-{D_0}^2\right)}{6N}\hfill \end{array}\right. $$

As donors can potentially transmit infection long after becoming infected (e.g. in the case of chronic infections) the distinction between past and future transmissions can be informative. This allows a decision maker to assess the impact of safety interventions and the necessity for communication of risks to transfusion recipients even at the end or (long) after an outbreak has occurred.

In situations where *D*_*i*_ < *D*_*v*_ and the infection occurs at the beginning of the visit, so within *D*_*v*_ < *D*_*i*_ after entering the risk area, the infectious period has already passed before the donor returns home and no transmission will occur. This means that the duration of exposure with a risk of transmission had length *D*_*i*_ instead of *D*_*v*_, and the risk of transmission has to be corrected for the likelihood of an early infection. The risk of the donor getting infected in this first period of his visit is equal to *λ*(*D*_*v*_ – *D*_*i*_). Hence the number of (future) infected donors can be calculated using Eqs. 1 and 3 whilst replacing the duration of the visit by the length of the infectious period (*D*_*i*_) and noting that the risk is overestimated by a (most likely very small) factor *1*/(*1* − *λ*(*D*_*v*_ − *D*_*i*_)).

The estimated number of infected donations might have to be adjusted according to the proportion of under-reporting of notified cases, according to the time that infected donors will actually donate during their infectious period before symptom onset (referred to as the critical infectious period in the original EUFRAT model), or adapted for the infectivity of the specific transfused product [2]. In addition, if more than one (infectious) product is derived from one donation, the number of transmissions will have to be adjusted accordingly.

### Application to outbreak data and sensitivity analysis

The travellers’ risk model was applied to the chikungunya outbreak in Italy in 2007 [2, 10]. We illustrate the risk for a group of non-Italian travellers, for instance from country A, who went to Italy during the outbreak and donate upon return in their home country. The analysis was done using the reported weekly number of infections in the outbreak region, corrected for the proportion of asymptomatic infection. To address the variation in incidence rates over time, the outbreak is modelled as a series of independent weekly outbreaks; thus the formulas were applied sequentially and the resulting estimated transmissions aggregated. To illustrate the sensitivity of the risk estimates to variation in model parameters, the numbers of transfusion transmissions were calculated for various values for the duration of stay and for the infectious period.

As a second example the Q fever outbreak, which lasted for over a 3-year period in the Netherlands, was assessed for the transmission risk of chronic Q fever in non-Dutch travellers to the Netherlands [12]. The monthly number of notified Q fever cases used in the analysis can be found in Additional file 1: Table S3. Note that the infectivity of chronic Q fever, in terms of pathogenicity and length of the infectious period, differs from that of the acute infection. Also, chronically infected travellers may be less likely to donate due to their underlying disease/condition (such as a heart valve abnormalities) [14, 15]. Nevertheless, we used chronic Q fever transmission to illustrate the risk from an infection with a long infectivity period. It is presumed that 2 % of the infections progress to a chronic infection with an infectious period of 12 months.

### Computer software

All formulas were derived using Mathematica (version 9.0.1, The Wolfram Centre Lower Road, Long Hanborough, Oxfordshire, United Kingdom). The formulas were implemented in Microsoft Excel (MS Office Professional Plus 2010, Excel version 14.0, Microsoft Corporation, One Microsoft Way Redmond, WA 98052–7329 USA) for analysis and graphing.

## Results

### Application to the chikungunya outbreak in Italy

**Fig. 2:**
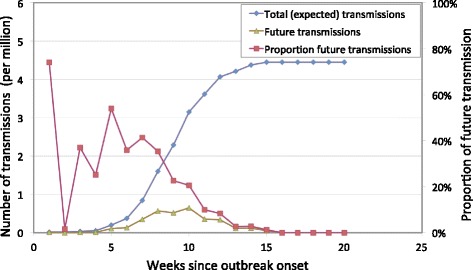
The estimated total cumulative number of transmissions, future transmissions and the corresponding proportion of future transmissions at the time considered. The estimates are based on the chikungunya outbreak in Italy in 2007 [2, 10]. The total number of expected transmissions (left y-axis) is estimated using the total number of cases notified up until that week (x-axis) of the outbreak. The number of expected future transmissions (left y-axis) is estimated using the same information, but also incorporates the timing of the occurrence of the infections. The proportion of future transmissions (right y-axis) is calculated as the ratio of future transmissions to the total number of transmissions estimated at the end of each week of the outbreak

Weekly chikungunya infections reported during the 15-week outbreak in 2007 in Italy were used to estimate the total number of transmissions by blood transfusion from travellers to Italy, as well as the expected proportion of future transmissions. The number of chikungunya infections was estimated by adjusting the notified cases for the proportion of asymptomatic cases (15 %); we used the adjusted value to allow for a comparison with earlier results. The expected future transmissions are shown in Table 2 and Fig. 2 for a scenario in which ten travellers per day (*τ* = 10) visited the risk area, remaining in the area for 7 days each (*D*_*v*_ = 7). For this scenario, the total cumulative (15 weeks) travellers’ transmission risk is 4.45 per million blood donations from travellers to Italy. At the end of outbreak week 7, the total number of expected transmissions is 0.85 per million and the proportion of future transmissions –given that 19 % of all infections were observed by the end of week 7– is 41 %. Implementation of interventions like stopping blood collection at this early stage only would substantially mitigate future transmissions from donors that travelled to the risk area.

**Table 2:** The weekly outbreak notified cases, estimated total number of transmissions by travelling donors, projected future transmissions and proportion of future transmissions resulting from current infections based on chikungunya outbreak data in Italy 2007 for a 7-day visit [2, 10]

Week number	Number of cases	Estimated cumulative total number of transmissions (per million)	Estimated future transmissions^a^ (per million)	Proportion of yet-to-occur transmissions (%)
*n*	*I*_*n*_	${N}_v(n)={\displaystyle \sum_{n=1}^{15}}\tau {D}_v\frac{I_n}{N}\varphi \left({D}_i-\frac{1}{2}{D}_v\right)$	${N}_{vf}(n)=\frac{\tau \varphi {I}_n\left(6{D}_v\left({D}_i-\frac{1}{2}{D}_v\right)-{D_0}^2\right)}{6N}$	$\frac{N_{vf}(n)}{N_v(n)}$
1	1	0.01	0.01	74
2	0	0.01	0.00	0
3	1	0.03	0.01	37
4	1	0.04	0.01	25
5	8	0.14	0.08	54
6	10	0.27	0.10	35
7	26	0.61	0.25	41
8	42	1.16	0.40	35
9	38	1.65	0.37	22
10	48	2.27	0.46	20
11	26	2.61	0.25	10
12	25	2.93	0.24	8.2
13	8	3.04	0.08	2.5
14	9	3.16	0.09	2.7
15	4	3.21	0.04	1.2

^a^Future transmissions were calculated using the formula given which estimates the number of transmission after the end of the corresponding week as a result of the observed cases in that week. As the infectious period lasts for 8 days, only 98 % of the future transmissions will actually occur in the week following the observed cases, and 2 % in the week after that. Therefore the number of future transmissions indicated in the table will on average underestimate the true number of future transmission by 2 %

### Sensitivity analysis

First, the duration of visit was varied. For duration of the travellers’ visit of 2, 4, and 8 days, the total transmission risk is equal to 2, 3.4 and 4.5 per million, respectively. Given that the infectious period is 8 days, the duration of the visit from 8 days onwards will result in a similar transmission risk for this setting. What can be observed is the decreasing relative increase in the number of transmissions with increasing visit length as a result of the negative quadratic term in visit length in Eq. 1. Here one can see that a four-fold increase in the duration of visit only doubles the risk. Therefore only for (relatively) short visits will the risk be linear in the duration of visit.

Next, the duration of visit was assessed for an infectious period of 1 year (365 days). Now the observed transmission risk becomes 100, 200, 410 and 710 per million for respective visit lengths of 2, 4, 8 and 14 days. Here it can be noted that the risk from travelling donors increases almost linear with the duration of visit (14/2 × 100 = 700 ≈ 710). The proportion of future transmissions at any given time during and after the outbreak period will be closely similar for each of these scenarios, as most transmissions (approximately 87 %) will occur after the end of the observed outbreak.

### Application to the Q fever outbreak in the Netherlands

**Fig. 3:**
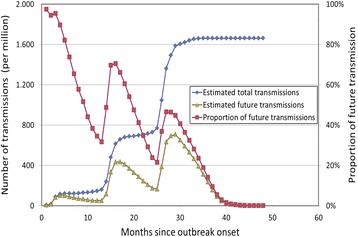
The estimated total cumulative number of transmissions, future transmissions and the corresponding proportion of future transmissions at the time considered. The estimates are based on the Q fever outbreak in The Netherlands in 2007–2009 [12]. The total expected number of transmissions (left y-axis) is estimated using the total number of cases notified up until that week (x-axis) of the outbreak. The number of expected future transmissions (left y-axis) is estimated using the same information, but also incorporates the timing of the occurrence of the infections. The proportion of future transmissions (right y-axis) is calculated as the ratio of future transmissions to the total number of transmissions estimated at the end of each week of the outbreak

Using the disease parameters from a previous Q fever study and visit characteristics as provided in Table 1 for analyses of the chikungunya outbreak, the number of transmissions from donors visiting the risk area in the Netherlands can be calculated. A constant stream of 10 visitors per day who on average stay for 2 weeks in the risk area would result in a total of 1.7 per thousand infection transmissions in the home country. Note that this number is derived directly from the number of notified cases (presuming 2 % chronic cases), whereas in the Q fever study the estimate was influenced by a proportion of more than 90 % undetected cases and a 50 % probability that symptomatic donors were screened out by the donor health questionnaire. The monthly outbreak analyses from the high-risk area indicated that at the annual outbreak peaks in 2007 (month 3, 36 cases), 2008 (month 15, 121 cases) and 2009 (month 27, 159 cases), the percentage of future transmissions are 95, 70 and 47 %, respectively, as shown in Fig. 3.

It is clear that incorporating uncertainty in all model parameters would allow construction of credible intervals (CIs) around the estimated number of transmissions. However, the figures are provided for illustration of the application of the methodology rather than for estimating the number of transmissions, hence CIs are not provided.

## Discussion

Our study shows that it is feasible to estimate the number of transmissions from travelling donors by applying the travellers’ risk model described in this paper. The model developed can support decision-making concerning safety interventions and communication related to donors travelling to EID risk areas. The model allows –using only a few basic parameters related to the outbreak, the infectious disease, and the travelling donor population– estimation of the transmission risk from travelling donors.

For the chikungunya outbreak we noted that the estimated traveller’s risk for a 1 week visit is 0.1 per million (0.1 infections in 1,000,000 donations made by travellers to the outbreak region). Liumbruno and colleagues reported that during this outbreak a 21-day deferral policy was implemented nationally for all donors who had visited the risk area even for a few hours, although their acceptable cut-off risk is one in 380,000, which is much higher than our model’s estimate [10]. Had the travellers’ risk model been available at that time, the policy might have been different. Chikungunya has only a short infectious period of 8 days. Therefore, only a limited number of future infections are anticipated. The ability of the travellers risk model to generate the expected number of future infections allows decision makers to quantify the potential immediate impact of (in)action on transfusion safety.

One of the limitations of our model is that it assumes that travelling donors only visit the risk area once. In some cases, travellers might make recurring visits and such repeated exposures might further increase their susceptibility to infection. This was also noted by Massad and colleagues [5]. For asymptomatic dengue infections repeat travel could expose a previously infected traveller to more severe expressions of illness, which might affect the donation behaviour of travellers. Data on such travel behaviour may not be easily available however. Another limitation of our model is that it presumes fixed donation intensity whereas donation behaviour is likely not to be constant in time, especially around periods of travel. When such information is available however, there are no restrictions to include these in modelling the risk of infected donations. Also, the current model presumes that the infection is transmissible immediately after infection. However, in case a disease is only infectious after a particular incubation time *D*_*c*_, the risk from such an infection can be calculated by simply calculating the number of infections that would have been transmitted if infections would have been transmitted over the full infectious period *D*_*i*_ and subtracting the infections transmitted from the time of infection up until the incubation time *D*_*c*_.

We noted that the travellers’ risk model is generally applicable, and its application is illustrated for two real outbreaks. Our model, with its distinction between past and future transmissions, can be considered a useful extension to currently available models for estimating transfusion transmission risks. Application of the travellers’ risk model may assist blood establishments in harmonising risks posed by travelling donors [9]. Several studies have shown that travellers do pose a risk [8, 16] which requires blood authorities to have mechanisms in place to manage such risks.

The example applications in this paper assumed 100 % transmissibility of the infection during the infectious period that will provide conservative risk estimates. The web based EUFRAT model however allows specification of the probability of transmission per type of transfusion product (which are typically red cells, platelets or plasma). It should be noted that transfusion transmission for chikungunya has not yet been demonstrated [10], and for Q fever the single suspected transmission case reported was not conclusively confirmed [12, 17]. Nevertheless, despite the unavailability of evidence concerning the transmission rate, decision-makers would still be interested in the expected number of transmissions for various viable scenarios. Such scenario analyses can only be conducted if models for calculating the transmission risks are available.

## Conclusions

We have developed a model that allows estimation of transfusion transmission risk from travelling donors. The limited number of generic model parameters required permits the model to be applied in very diverse settings. This not only empowers public health decision-makers with an appropriate tool to objectively quantify the risk from traveling donors, but also provides a sound proactive basis for enhanced management and response to outbreak situations.

## Additional file

Additional file 1: Deriving the risk of transfusion transmission from travelling donors. (DOCX 91 kb)

## Abbreviations

EID: emerging infectious diseases
TT: transfusion transmission
