# Supplementary material for: Modelling the risk of transfusion transmission from travelling donors
Source: BMC Infect Dis. 2016 Apr 1;16:143. doi: 10.1186/s12879-016-1452-z (PMC4818889; doi:10.1186/s12879-016-1452-z)
Supplement: Additional file 1: — Deriving the risk of transfusion transmission from travelling donors. (DOCX 91 kb) [file 12879_2016_1452_MOESM1_ESM.docx]

# Additional files

# Appendix: Deriving the risk of transfusion transmission from travelling donors

***Modelling assumptions and the total number of transfusion transmissions***

We consider the situation that a travelling donor enters an area with ongoing transmission (we call it the risk area) at time $t_{e}$, he remains there for a time period $D_{v}$ and then returns to his home country. If he becomes infected during his visit and upon returning home donates blood whilst still being infectious, transmission through infected blood products will occur.

Now assume that we know the incidence *i*(*t*) of the infection in the risk area in a time interval [$0,t_{obs}$] of length $D_{0}$. Assume also that the incidence can be interpreted as a proxy for the force of infection to which a susceptible traveller is exposed; more specifically, we assume that $\lambda\left( t \right)=c i(t)$ with some constant factor $c$. We assume that the time of entry of the travelling donor into the area $t_{e}$ is uniformly distributed over the time interval [$-D_{v},t_{obs}$].

We define

$\lambda\left( t \right)=$ $\left\{ \begin{matrix} 0 & \mathrm{for}t\leq0 \\ c i(t) & \mathrm{for}0<t\leq t_{obs} \\ 0 & \mathrm{for}t>t_{obs} \end{matrix} \right.$ (*Eq.A1*)

Then the probability that the travelling donor will be infected during his stay in the risk area is given by

$P_{inf}\left( t_{e} \right)=\int_{t_{e}}^{t_{e}+D_{v}} \text{λ(}t_{i}\text{)}\text{d}t_{i}=\int_{max(t_{e},0)}^{{min(t}_{e}+D_{v},D_{o})} \text{λ(}t_{i}\text{)}\text{d}t_{i}$ (*Eq.A2*)

For a constant $\lambda$ in [$0,t_{obs}$] the risk of a traveling donor becoming infected who enters the risk area at time point $t_{e}$ is simply $\lambda\left( \min\left( D_{o},t_{e}+D_{v} \right)-\max\left( t_{e},0 \right) \right)$, where $-D_{v}{\leq t}_{e}\leq D_{o}$. There will be a gradual increase in risk until it reaches a plateau, after which there will be a decrease until 0 again. The exact change points and height of the risk will be dependent on the relative lengths of $D_{o}$ and $D_{v}$.

The time of infection $t_{i}$ lies in the interval [$t_{e},t_{e}+D_{v}$]. We assume that the travelling donor is infectious for a fixed period $D_{i}$, which means the time interval of infectivity is [$t_{i},t_{i}+D_{i}$]. However, as the travelling donor only starts donating after his return home, an infected donation can only occur in the time interval [$t_{e}+D_{v}, t_{i}+D_{i}$]. We presume that the donation rate is constant, which means that the donation risk is proportional to the length of the interval [$t_{e}+D_{v}, t_{i}+D_{i}$]. We denote the donation rate by $\varphi$. If travelling donors enter the risk area at a constant rate $\tau$ and remain there for a time period $D_{v}$ we can compute the number of infected donations from travelling donors ($N_{v}$) as:

$$N_{v}=\int_{t_{e}=-D_{v}}^{D_{o}} \int_{t_{i}=Max(t_{e} , 0)}^{Min(D_{o}{, t}_{e}+D_{v})} \int_{t_{x}=t_{e}+D_{v}}^{t_{i}+D_{i}} \tau\lambda\varphi dt_{x} dt_{i}dt_{e}$$

$=\tau\lambda\varphi D_{o}\left( D_{i}-½D_{v} \right)D_{v}$ (*Eq.A3*)

Basically this is just the sum of all possible combination of a donor entering the risk area at time $t_{e}$ ($\tau dt_{e}$), this donor getting infected and starts being infectious at time $t_{i}$ ($\lambda dt_{i}$), and this donor donating at time $t_{x}$ ($\varphi dt_{x}$).

Assume a constant incidence rate $\lambda=\frac{I}{ND_{0}}$, where *I* is the number of infections notified, *N* is the size of the population at risk and $D_{0}$ equals the length of the observed outbreak. Then, with the donation rate $\varphi$ being one over the time interval between subsequent donations ($D_{d}$), then *Equation A3* can be re-written as:

$N_{v}=\frac{D_{v}\tau}{N}I\frac{\left( D_{i}-½D_{v} \right)}{D_{d}}$ (*Eq.A4*)

***Derivation of future transmissions for*** $\boldsymbol{D}_{\boldsymbol{i}}\boldsymbol{\geq}\boldsymbol{D}_{\boldsymbol{v}}$

If $t_{e}+D_{v}<D_{o}$ *Equation A3* can be split up into infected donations that have already been made before $t_{obs}$ (past transmissions), and those projected to occur after$t_{obs}$ (future transmissions). An expression for the number of future transmissions from travelling donors can be derived by adding an indicator function $\left( \mathbb{1}_{\left\{ condition \right\}} \right)$to the integral. The indicator function is a function that has the value 1 whenever the subscript $\left\{ condition \right\}$ is fulfilled and is equal to 0 if this is not the case. Therefore by adding the indicator function $\mathbb{1}_{\left\{ t_{x}>t_{obs} \right\}}$ to equation *3* would allow calculating the number of future transfusion transmission as only donations that would occur beyond $t_{obs}$ would contribute to the summation. In a similar fashion the condition that infections can occur only during the overlap of visit and actual outbreak, which is now implemented by means of restrictions in the boundaries of the integral for $t_{i}$, can also be enforced by an indicator function. The number of future transmissions by travelling donors expected after time point $t_{obs}$ can therefore (for $D_{i}\geq D_{v}$) be calculated from:

$N_{vf}=\int_{t_{e}=-D_{v}}^{D_{o}} \int_{t_{i}=t_{e}}^{t_{e}+D_{v}} \int_{t_{x}=t_{e}+D_{v}}^{t_{i}+D_{i}} \tau\lambda\mathbb{1}_{\left\{ 0<t_{i}<t_{obs} \right\}} \varphi\mathbb{1}_{\left\{ t_{x}>t_{obs} \right\}}{dt_{x}dt_{i}dt}_{e}$ *(Eq.A5*)

The outcome of this integral depends on the lengths of $D_{v}$, $D_{i}$, and $D_{o}$. There are three combinations where the duration of the infectious period is longer than the duration of visit ($D_{i}\geq D_{v}$), as illustrated in **Appendix Figure 1**. The expressions for the number of future transmissions from infected donors ($N_{vf}$) for these situations are:

$N_{vf}=\left\{ \begin{matrix} D_{0}\geq D_{i}\geq D_{v}: & \frac{\tau I\varphi\left( 3D_{i}^{2}-{D_{v}}^{2} \right)D_{v}}{6ND_{0}} \\ D_{i}\geq D_{0}\geq D_{v}: & \frac{\tau I\varphi\left( 6D_{0}D_{i}-3{D_{0}}^{2}-{D_{v}}^{2} \right)D_{v}}{6ND_{0}} \\ D_{i}\geq D_{v}\geq D_{0}: & \frac{\tau I\varphi\left( 6D_{v}D_{i}-3{D_{v}}^{2}-{D_{0}}^{2} \right)}{6N} \end{matrix} \right.$  *(Eq.A6*)


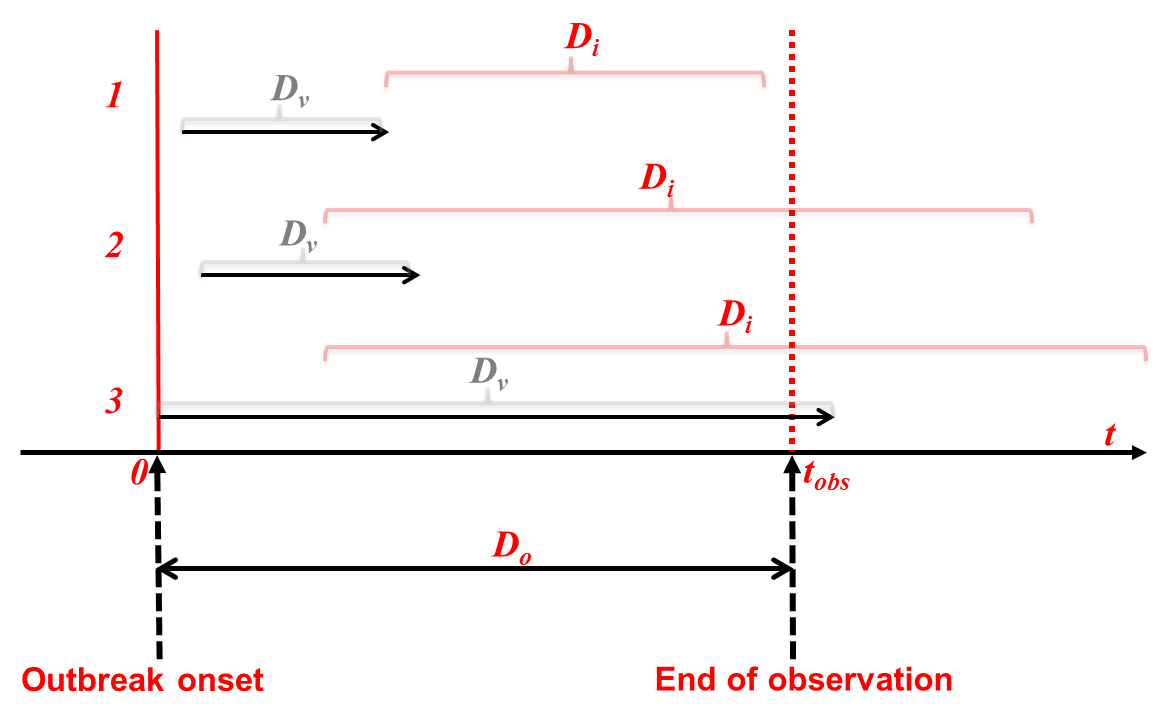


**Appendix Figure 1- Illustration of different settings applicable to each of the formulas in *Equation A5*, in which the duration of visit** $\boldsymbol{(}\boldsymbol{D}_{\boldsymbol{v}}$**), duration of infectious period** $\boldsymbol{(}\boldsymbol{D}_{\boldsymbol{i}}$**), and duration of the observed outbreak** $\boldsymbol{(}\boldsymbol{D}_{\boldsymbol{o}}$**) vary (for** ${\boldsymbol{D}_{\boldsymbol{i}}\boldsymbol{\geq D}}_{\boldsymbol{v}}$**). Setting 1:** $\boldsymbol{D}_{\boldsymbol{o}}\boldsymbol{\geq}\boldsymbol{D}_{\boldsymbol{i}}\boldsymbol{\geq}\boldsymbol{D}_{\boldsymbol{v}}$**, Setting 2:** $\boldsymbol{D}_{\boldsymbol{i}}\boldsymbol{\geq}\boldsymbol{D}_{\boldsymbol{0}}\boldsymbol{\geq}\boldsymbol{D}_{\boldsymbol{v}}$**, and Setting 3:** $\boldsymbol{D}_{\boldsymbol{i}}\boldsymbol{\geq}\boldsymbol{D}_{\boldsymbol{v}}\boldsymbol{\geq}\boldsymbol{D}_{\boldsymbol{o}}$**.**

Since the total number of transmissions $N_{v}$ by travelling donors is estimated by $\tau I{\varphi D}_{v}\left( D_{i}-½D_{v} \right)/N$, the number of future transmissions can be re-written as a proportion of the total number of transmissions. This proportion of future transmissions gives insight in the magnitude of the transmission risk that can still be prevented. The formulas for the proportion of future transmissions ($P_{vf}$) are:

| $P_{vf}=\left\{ \begin{matrix} D_{0}\geq D_{i}\geq D_{v}: & \frac{\left( ½D_{i}^{2}-⅙{D_{v}}^{2} \right)}{D_{0}(D_{i}-½D_{v})} \\ D_{i}\geq D_{0}\geq D_{v}: & \frac{\left( D_{0}\left( D_{i}-½D_{0} \right)-⅙{D_{v}}^{2} \right)}{D_{0}(D_{i}-½D_{v})} \\ D_{i}\geq D_{v}\geq D_{0}: & 1-\frac{{⅙D_{0}}^{2}}{D_{v}(D_{i}-½D_{v})} \end{matrix} \right.$ | (*Eq.A7*) |
| --- | --- |

For $D_{0}\geq D_{i}\geq D_{v}$, the proportion of future transmissions lies between 0 and ⅔ of all transmissions. The proportion of future transmissions is determined by the ratio of $D_{i}$ and $D_{0}$: for (relatively) small values of $D_{v}$ the proportion of future transmissions becomes $½D_{i}/D_{0}$ of all transmissions, for large values of $D_{v}$ this proportion increases to $⅔D_{i}/D_{0}$ of all transmissions.

Between 50% and 100% of all transmissions will occur after $t_{obs}$ in case $D_{i}\geq D_{0}\geq D_{v}$. It is clear that for small values of$D_{0}$ and $D_{v}$ as compared to $D_{i}$the vast majority of transmissions will occur in future. However, the smallest proportion of future transmissions (around 50%) will occur in future when $D_{v}$ is small compared to both $D_{0}$ and $D_{i}$, whenever the latter two are of comparable magnitude.

In case $D_{i}\geq D_{v}\geq D_{0}$ the majority of transmissions (more than two-thirds) will occur in future. Whenever $D_{i},D_{0}$ and $D_{v}$ are roughly of the same magnitude two-thirds of all transmissions are expected to occur in future. Relatively small values of $D_{0}$ will obviously result in a large number of future transmissions. It can be found that the number of future infections reduces not only with the ratio of the duration of the outbreak and the infectious period (${D_{0}/D}_{i}$, which here has to be corrected for the visiting time), but now also for the ratio of the duration of the outbreak and visiting time ($D_{0}/D_{v}$).

**Derivation of resident donors’ transfusion transmission risk**

Similar to the derivation of the risk of transfusion transmission from travellers, formulas for estimating the risk to donors residing in the risk area can be derived. We include this derivation here because such a formula is not available in literature, but nevertheless might be helpful for decision-making support in the risk area itself.

Assume a population of size$N$ in which an outbreak occurs which has lasted for a time period $D_{0}$, and which has resulted in$I$ infections. If we assume that the incidence rate (= the number of infections per unit time) is constant over the outbreak period, then the incidence rate ($\lambda$) is equal to $\frac{I}{ND_{o}}$. Donors (a proportion $p_{d}$ of the population) are continuously exposed (as any other member of the general population), and donate with a donation interval of $D_{d}$. The donation rate of a donor at any point in time is $\varphi=\frac{1}{D_{d}}$. After becoming infected, a donor is infectious for a set period $D_{i}$.

At any time point $t$ during the outbreak (so in the time interval $[0,t_{obs}]$ with length $D_{o}$), the likelihood that a donor gets infected is equal to ${Np}_{d}\lambda$. For any donor who gets infected at time point $t_{i}$ at any time point $t_{x}$ in the interval $\left[ t_{i},t_{i}+D_{i} \right]$, the donor will have a probability of $\varphi$ of delivering an infected donation. The total number of infected resident donors’ transmissions ($N_{r}$) can therefore be calculated as follows:

| $N_{r}=\int_{t_{i}=0}^{D_{o}} \int_{t_{x}=t_{i}}^{t_{i}+D_{i}} {N p}_{d} \lambda\varphi{{dt}_{x}dt}_{i}$  $={Np}_{d}\lambda\varphi D_{0}D_{i}={Np}_{d}\frac{I}{ND_{0}}\varphi D_{0}D_{i}=p_{d}I\frac{D_{i}}{D_{d}}$ | (*Eq.A8*) |
| --- | --- |

The number of infected donations is simply the total number of infections multiplied by the proportion of donors multiplied by the expected number of donations by an infected donor, which is equal to the ratio of infectious period and inter-donation interval.

To estimate the number of future transmissions, only transmissions at time points $t_{x}$ during the time interval $D_{t}$ that lies beyond $t_{obs}$ have to be counted. This can be achieved by adding an indicator function to the integral to account for this condition. The number of future transmissions from resident donors ($N_{rf}$) can be calculated as follows:

| $N_{rf}=\int_{t_{i}=0}^{D_{o}} \int_{t_{x}=t_{i,}}^{t_{i}+D_{i}} {N p}_{d} \lambda f_{d}\mathbb{1}_{\left\{ t_{x}>D_{o} \right\}} dt_{x}dt_{i} =\left\{ \begin{matrix} D_{0}\geq D_{i}: & \frac{p_{d}ID_{i}}{D_{d}}\cdot\frac{{½D}_{i}}{D_{0}} \\ D_{0}\leq D_{i}: & \frac{p_{d}ID_{i}}{D_{d}}\cdot\left( 1-\frac{½D_{0}}{D_{i}} \right) \end{matrix} \right.$ | (*Eq.A9*) |
| --- | --- |

From *Equation A9* it becomes clear that for $D_{0}\geq D_{i}$ less than half the transmissions will occur in the future, and that this proportion will depend on the ratio of the infectious period ($D_{i}$) to the duration of the outbreak ($D_{0}$). In the case $D_{0}\leq D_{i}$ the reverse will be true, and the majority of transmissions is expected to occur in the future. The exact number of future transmissions will again depend on the ratio of $D_{i}$ and $D_{0}$.

**Appendix table 1**- The monthly outbreak notified cases, estimated total number of transmissions by travelling donors, projected future transmissions and proportion of future transmissions resulting from current infections based on Q fever outbreak data in the Netherlands 2007-2009 for a 7-day visit.^13^

| **Month number** | **Number of acute cases** | **Number of chronic cases (2% of acute cases)** | **Estimated cumulative total number of transmissions**  **(per million)** | **Estimated future transmissions***  **(per million)** | **Proportion of yet-to-occur transmissions ****  **(%)** |
| --- | --- | --- | --- | --- | --- |
| *n* |  | $I_{n}$ | $N_{v}(n)=\sum_{n=1}^{35} \tau D_{v}\frac{I_{n}}{N}\varphi\left( D_{i}-\frac{1}{2}D_{v} \right)$ | $N_{vf}(n)$* | $\frac{N_{vf}\left( n \right)}{N_{v}\left( n \right)}$ |
| 1 | 3 | 0.06 | 6 | 6 | 97 |
| 2 | 5 | 0.1 | 16 | 15 | 94 |
| 3 | 36 | 0.72 | 87 | 83 | 95 |
| 4 | 14 | 0.28 | 115 | 103 | 90 |
| 5 | 3 | 0.06 | 121 | 100 | 82 |
| 6 | 0 | 0 | 121 | 89 | 74 |
| 7 | 0 | 0 | 121 | 79 | 65 |
| 8 | 1 | 0.02 | 123 | 71 | 58 |
| 9 | 3 | 0.06 | 129 | 67 | 52 |
| 10 | 1 | 0.02 | 131 | 58 | 44 |
| 11 | 3 | 0.06 | 137 | 53 | 38 |
| 12 | 5 | 0.1 | 147 | 51 | 35 |
| 13 | 6 | 0.12 | 159 | 50 | 32 |
| 14 | 41 | 0.82 | 240 | 117 | 49 |
| 15 | 121 | 2.42 | 481 | 335 | 70 |
| 16 | 67 | 1.34 | 614 | 433 | 71 |
| 17 | 23 | 0.46 | 659 | 436 | 66 |
| 18 | 11 | 0.22 | 681 | 413 | 61 |
| 19 | 3 | 0.06 | 687 | 372 | 54 |
| 20 | 2 | 0.04 | 691 | 328 | 47 |
| 21 | 4 | 0.08 | 699 | 288 | 41 |
| 22 | 2 | 0.04 | 703 | 245 | 35 |
| 23 | 6 | 0.12 | 715 | 208 | 29 |
| 24 | 7 | 0.14 | 729 | 174 | 24 |
| 25 | 21 | 0.42 | 770 | 166 | 22 |
| 26 | 139 | 2.78 | 1 046 | 386 | 37 |
| 27 | 159 | 3.18 | 1 362 | 634 | 47 |
| 28 | 66 | 1.32 | 1 493 | 693 | 46 |
| 29 | 46 | 0.92 | 1 585 | 710 | 45 |
| 30 | 10 | 0.2 | 1 604 | 652 | 41 |
| 31 | 9 | 0.18 | 1 622 | 592 | 37 |
| 32 | 9 | 0.18 | 1 640 | 532 | 32 |
| 33 | 7 | 0.14 | 1 654 | 466 | 28 |
| 34 | 3 | 0.06 | 1 660 | 392 | 24 |
| 35 | 1 | 0.02 | 1 662 | 314 | 19 |
| 36 | 0 | 0.00 | 1 662 | 235 | 14 |
| 37 | 0 | 0.00 | 1 662 | 158 | 10 |
| 38 | 0 | 0.00 | 1 662 | 91 | 5 |
| 39 | 0 | 0.00 | 1 662 | 49 | 3 |
| 40 | 0 | 0.00 | 1 662 | 27 | 2 |
| 41 | 0 | 0.00 | 1 662 | 15 | 1 |
| 42 | 0 | 0.00 | 1 662 | 9 | 1 |
| 43 | 0 | 0.00 | 1 662 | 5 | 0 |
| 44 | 0 | 0.00 | 1 662 | 2 | 0 |
| 45 | 0 | 0.00 | 1 662 | 1 | 0 |
| 46 | 0 | 0.00 | 1 662 | 0 | 0 |

* Future infections for the situation where $D_{i}\geq D_{0}\geq D_{v}$ (365 ≥ 30 ≥ 14) can be calculated as: $N_{vf}\left( n \right)=\frac{\tau I_{n}\varphi\left( 6D_{0}D_{i}-3{D_{0}}^{2}-{D_{v}}^{2} \right)D_{v}}{6ND_{0}}$ (main paper, equation 3). However, this number refers to the total number of estimated transmissions after the 30 days observation interval. The total number of infections after interval $n$ however requires addition of expected transmissions from all previous intervals.

Define the function $P_{vf}\left( D_{0}, D_{i},D_{v} \right)$ that returns the proportion of future outbreaks (so after time point $D_{0}$) from travelling donors. This function is dependent on the duration of the outbreak, the duration of the acute infection and the duration of stay in the outbreak area:

| $P_{vf}\left( D_{0}, D_{i},D_{v} \right)=\left\{ \begin{matrix} \forall\left( D_{0}\geq D_{i}\geq D_{v} \right): & \frac{\left( ½D_{i}^{2}-⅙{D_{v}}^{2} \right)}{D_{0}\left( D_{i}-½D_{v} \right)} \\ \forall\left( D_{i}\geq D_{0}\geq D_{v} \right) : & \frac{\left( D_{0}\left( D_{i}-½D_{0} \right)-⅙{D_{v}}^{2} \right)}{D_{0}\left( D_{i}-½D_{v} \right)} \\ \forall\left( D_{i}\geq D_{v}\geq D_{0} \right) : & 1-\frac{{⅙D_{0}}^{2}}{D_{v}\left( D_{i}-½D_{v} \right)} \end{matrix} \right.$ |
| --- |

*(Eq.A10)*

With this function, the proportion of infections after any point in time $t$ can be calculated as:

| $P_{tvf}\left( D_{0}, D_{a},D_{v},t \right)=$  $\left\{ \begin{matrix} \forall\left( t<0 \right): & 1 \\ \forall\left( 0\leq t\leq D_{0} \right): & \left( 1 -\frac{t}{D_{0}} \right)+ \frac{t}{D_{0}}*P_{vf}\left( t, D_{a},D_{v} \right) \\ \forall\left( D_{0}<t \right): & \frac{t}{D_{0}}*P_{vf}\left( t, D_{a},D_{v} \right)-\left( \frac{t}{D_{0}}-1 \right)*P_{vf}\left( t-D_{0}, D_{a},D_{v} \right) \end{matrix} \right.$ |
| --- |

*(Eq.A11)*

The rationale here is that for any $t$ less than $D_{0}$the interval $D_{0}$can be split in two, one with length $t$ and one with length$\left( D_{0}-t \right)$. The total number of transmissions from either interval will be proportional to its length. Infections obtained in the second interval, which is equal to a proportion $\left( 1 -\frac{t}{D_{0}} \right)$ of all infections, will all be transmitted beyond time point $t$ as donors will all obtain these infections beyond time point $t$. Of the proportion of infections that occurred before time point $t \left( \frac{t}{D_{0}} \right)$the proportion of infections transmitted after time point $t$ can be calculated as $P_{vf}\left( t, D_{a},D_{v} \right)$, simply presuming an outbreak of length $t$. To calculate the number of transmissions beyond time point $D_{0}$the duration of the outbreak is presumed to be extended to time point $t$ and therefore transmitting $\frac{t}{D_{0}}$ as many infections in total. Next, the number of transmissions beyond $t$ can be calculated as $\frac{t}{D_{0}}*P_{vf}\left( t, D_{a},D_{v} \right)$ which is the increase of the number of transmissions from the extended outbreak $\left( \frac{t}{D_{0}} \right)$ times the proportion of infections beyond $t$ $\left( P_{vf}\left( t, D_{a},D_{v} \right) \right)$ minus the number of infections beyond time point $t$ that arise from the extended outbreak that never occurred $\left( \left( \frac{t}{D_{0}}-1 \right)*P_{vf}\left( t-D_{0}, D_{a},D_{v} \right) \right)$.
